# A Novel Nomogram for Prediction and Evaluation of Lymphatic Metastasis in Patients With Renal Cell Carcinoma

**DOI:** 10.3389/fonc.2022.851552

**Published:** 2022-04-11

**Authors:** Wenle Li, Bing Wang, Shengtao Dong, Chan Xu, Yang Song, Ximin Qiao, Xiaofeng Xu, Meijin Huang, Chengliang Yin

**Affiliations:** ^1^ Clinical Medical Research Center, Xianyang Central Hospital, Xianyang, China; ^2^ Department of Spine Surgery, Second Affifiliated Hospital of Dalian Medical University, Dalian, China; ^3^ Department of Gastroenterology and Hepatology, Chinese People’s Liberation Army (PLA) General Hospital, Beijing, China; ^4^ Department of Urology, Xianyang Central Hospital, Xianyang, China; ^5^ Department of Oncology, 920th Hospital of People's Liberation Army (PLA) Joint Logistics Support Force, Yunnan, China; ^6^ Faculty of Medicine, Macau University of Science and Technology, Macau, Macau SAR, China

**Keywords:** nomogram, renal cell carcinoma (RCC), lymphatic metastasis, multicenter, web calculator

## Abstract

**Background:**

Lymphatic metastasis is an important mechanism of renal cell carcinoma (RCC) dissemination and is an indicator of poor prognosis. Therefore, we aimed to identify predictors of lymphatic metastases (LMs) in RCC patients and to develop a new nomogram to assess the risk of LMs.

**Methods:**

This study included patients with RCC from 2010 to 2018 in the Surveillance, Epidemiology, and Final Results (SEER) database into the training cohort and included the RCC patients diagnosed during the same period in the Second Affiliated Hospital of Dalian Medical University into the validation cohort. Univariate and multivariate logistic regression analysis were performed to identify risk factors for LM, constructing a nomogram. The receiver operating characteristic (ROC) curves were generated to assess the nomogram’s performance, and the concordance index (C-index), area under curve value (AUC), and calibration plots were used to evaluate the discrimination and calibration of the nomogram. The nomogram’s clinical performance was evaluated by decision curve analysis (DCA), probability density function (PDF) and clinical utility curve (CUC). Furthermore, Kaplan-Meier curves were performed in the training and the validation cohort to evaluate the survival risk of the patients with lymphatic metastasis or not. Additionally, on the basis of the constructed nomogram, we obtained a convenient and intuitive network calculator.

**Results:**

A total of 41837 patients were included for analysis, including 41,018 in the training group and 819 in the validation group. Eleven risk factors were considered as predictor variables in the nomogram. The nomogram displayed excellent discrimination power, with AUC both reached 0.916 in the training group (95% confidence interval (CI) 0.913 to 0.918) and the validation group (95% CI 0.895 to 0.934). The calibration curves presented that the nomogram-based prediction had good consistency with practical application. Moreover, Kaplan-Meier curves analysis showed that RCC patients with LMs had worse survival outcomes compared with patients without LMs.

**Conclusions:**

The nomogram and web calculator (https://liwenle0910.shinyapps.io/DynNomapp/) may be a useful tool to quantify the risk of LMs in patients with RCC, which may provide guidance for clinicians, such as identifying high-risk patients, performing surgery, and establishing personalized treatment as soon as possible.

## Introduction

Renal cell carcinoma (RCC) is the most common malignant tumor of the kidney, ranking sixth in men and tenth in women, accounting for 5% to 3% of all tumors (1). The most common subtype of RCC is clear cell RCC, which accounts for approximately 70-80%. Other subtypes include papillary RCC (pRCC, 10-15%) (2), chromophobic RCC (chRCC, 5-10%) (3), the rare collecting duct RCC (cdRCC) and sarcomatoid RCC (srRCC)) (4). According to the latest report, more than 140,000 people die from RCC yearly, ranking the 13th most common cause of cancer death worldwide (5). With the improvement of examination methods, despite the fact that most of the lesions are found to be small, there are still a considerable number of patients diagnosed as locally advanced stage, and up to 17% of patients had distant metastases (6). The most common metastatic sites of RCC involve lung, lymph nodes, liver, bone and adrenal glands. Among them, local lymph node metastasis is a most important adverse prognostic factors for adult RCC, resulting in an 8-fold risk of death (7, 8). Therefore, it is critical for clinicians to accurately evaluate the risk of lymph node metastasis and formulate the optimal treatment plan. Anatomically, the lymphatic drainage structure of the kidney is complex, variable and inconsistent, making the discovery, diagnosis, and evaluation of LMs in RCC particularly difficult. As we all know, computed tomography (CT) and magnetic resonance (MRI) are currently the ideal tools for the diagnosis and staging of RCC, but they cannot accurately predict LMs, and their ability to distinguish normal size or micrometastasis is limited (9). In addition to the unclear imaging findings, the low positive rate of intraoperative biopsy can also lead to the failure of detection and diagnosis of lymphatic metastasis in RCC early, thereby limiting the therapeutic effect. Thus, improving the awareness and monitoring of LMs will contribute to improve the prognosis of RCC patients. However, there is currently no relevant research focused on developing an ideal predictive model to predict the risk of LMs in RCC, which means that the probability of occurrence of LMs cannot be quantified. Recently, nomogram is a novel type of prognostic tool, which is widely used in oncology and medicine to help clinicians predict prognosis and make medical decisions (10–14). Therefore, we utilized the Surveillance, Epidemiology, and End Results (SEER) database, which is often used to study rare tumors. The database provides data from 18 cancer registries, including approximately 30% of the U.S. population. To address this, by integrating different clinical variables, our study first developed a nomogram to predict LMs in RCC patients to provide an individual risk assessment and medical decision-making for patients.

## Methods

### Study Design and Participants

Patients diagnosed with RCC between 2010 and 2018 from the SEER database through the SEER&STAT software (version 8.3.9.2) were collected and the patients who met the following inclusion criteria were grouped into a training group. Patients diagnosed in the Second Affiliated Hospital of Dalian Medical University at the same time as the SEER database were included in the external verification group.

The inclusion criteria were as follows: (1) the patient was older than 18 years, (2) patients with primary kidney cancer (International Classifification of Diseases for Oncology ICD-O. 8120/3 represents transitional cell carcinoma, 8130/3 represents papillary transitional cell carcinoma, 8260/3 represents papillary adenocarcinoma, 8310/3 represents clear cell adenocarcinoma, 8312/3 represents renal cell carcinoma, 8317/3 represents chromophobe renal cell carcinoma) diagnosed between January 1, 2010 and December 31, 2017, (3) no previous or concurrent history of other malignant tumors, (4) according to the 8th American Joint Committee on Cancer (AJCC) TNM Staging Manual, re-staging the enrolled patients, and (5) there were sufficient imaging and pathological results during the follow-up period to assess whether the metastasis was in progress happen.

The exclusion criteria were as follows: (1) patients younger than 18, (2) multiple malignant tumor history or the same period, (3) unable to obtain complete demographic characteristics, including age, gender, race, etc., (4) unable to obtain tumor information, including size, stage, histological type, TNM stage, etc.,(5) diagnosis was from cadavers, (6) with unknown LMss and survival time, and (7) cause of death unrelated to RCC or unknown.

This study was approved by the institutional ethics committee.

### Data Collection

A total of 41837 RCC patients fulfilled the inclusion criteria were incorporated into the final analyses. All data of the training group were obtained from the SEER database, and the data of the verification group were obtained from the Second Affiliated Hospital of Dalian Medical University. Fifteen variables that might be related to the development of LMs in RCC patients were included in the study. Demographic characteristics and clinical variables included age, race, gender, marriage, One primary only or more, primary site, the degree of tumor differentiation, tumor size, histological type, T stage, M stage, with or without LMs, alive or not. The data of the verification group were collected by two researchers and one researcher was responsible for verification. Tumor-related information is provided by clinicians, and pathological information is diagnosed by two pathologists using a double-blind method and reviewed by a senior pathologist.

### Construction of Nomogram and Statistical Analysis

R language (version 4.0.5) and SPSS 25.0 were used for all statistical analyses in this study. The prediction nomogram was constructed based on the patients in the training group and tested by the patients in the validation group. The independent sample t test was utilized to analyze continuous variables, and the chi-square test was used to analyze categorical variables. Univariate logistic analysis was carried out to identify LMs-associated risk factors. Variables with a *P* value < 0.05 in univariate analysis were further incorporated into multivariate logistic regression analysis to identify the independent risk factors for LMs in RCC patients. Significant independent risk factors verified by multivariate logistic regression analysis were used to construct nomogram with the “rms” package in R software. Receiver operating characteristic (ROC) curve was drawing by medcalc to test the performance of the predictive model, and area under the curve (AUC) was used to express the recognition ability of the predictive model. The area was larger, the recognition ability was better. Probability density function (PDF) was plotted to identify the key points suitable for clinical application, and clinical utility curve (CUC) was used to compare the clinical benefits under different thresholds. Additionally, the consistency of the model was verified by drawing a calibration curve. The decision curve analysis (DCA) was used to verify and evaluate its clinical applicability. Meanwhile, we also performed Kaplan-Meier survival analysis on the overall survival rate of the included patients and used the log-rank test to determine the significance of the difference between the internal and external cohort survival curves. Furthermore, based on the constructed nomogram, we also provide a convenient and intuitive web calculator. Statistical analysis was performed using SPSS (version 20.0, Chicago, IL, USA). A p-value < 0.05 (two-tailed) was considered statically significant. The R language software packages applied for developing predictive model included plyr, rms, foreign, DynNom, regplot, caret, ggDCA, ggpubr, pROC, patchwork, eoffice, gLMsnet, survival.

## Results

### Basic Characteristics of Patients

A total of 41837 RCC patients diagnosed from 2010 to 2017 were enrolled in this study, of which 41018 patients from the SEER database were included in the training group, and 819 patients from the Second Affiliated Hospital of Dalian Medical University were included in the verification group. The demographic and clinical characteristics of the two groups were collected in **Table 1**. In the training and validation groups, the age of the patients ranged from 55 to 75 years, with a mean age of 64, 65 years, respectively. Most of the patients were male, and the ratio of male to female was roughly similar in the two groups. In the training group, most of them were white (78.11%), and only 1.2% were Chinese. The majority of the patients (58.86% and 65.57%, respectively) were married. The most common histologic subtype was clear cell adenocarcinoma (8310/3) (53.36% and 55.68%, respectively). Primary tumor location was mainly in the kidney, and the degree of differentiation was mostly moderately differentiated, accounting for 34.70% and 37.36% respectively. According to the guidelines of the American Joint Committee on Cancer (AJCC), the most common T stage was T1 (66.11% and 60.68%, respectively). Moreover, the whole population had a relatively low rate of lymph node metastasis, occurred in 2630 (6.41%) patients in the training set and 66 (8.06%) patients in the validation set. In the two groups, only 11.04% and 14.53% of the patients presented with metastatic tumors at diagnosis, respectively. Most of the patients were alive during the follow-up period (74.33% and 73.50%, respectively). There were no statistically significant differences in the lymph node metastasis rate, age, one primary only or more, time, alive or dead, sex and tumor size between the two groups (P>0.05). However, there were statistical differences in M-stage, marital status, race, primary site, grade, laterality, pathological and T-stage of the the training and validation groups (P<0.05). Additionally, according to the presence or absence of LMs in RCC, all patients were divided into two subgroups: lymph node metastasis negative (LNN=39141) and lymph node metastasis positive (LNP=2696). The difference between the two subgroups was shown in **Table 2**. With the exception of race, variables differed significantly between the two subgroups.

**Table 1:** Baseline of patients in the training and validation groups.

Characteristics	Level	Training group (N=41018)	Validation group (N=819)	p
Lymph.node.metastasis (%)	No	38388 (93.59)	753 (91.94)	0.0675
	Yes	2630 (6.41)	66 (8.06)	
M (%)	M0	36490 (88.96)	700 (85.47)	0.002
	M1	4528 (11.04)	119 (14.53)	
Marital (%)	Married	24143 (58.86)	537 (65.57)	<0.0001
	unknown	2002 (4.88)	0 (0.00)	
	unmarried	14873 (36.26)	282 (34.43)	
Age (median [IQR])	not available	64.000 [55.000, 73.000]	65.000 [55.000, 73.000]	0.383
Race.ethnicity (%)	black	5225 (12.74)	0 (0.00)	<0.0001
	Chinese	492 (1.20)	819 (100.00)	
	other	3263 (7.96)	0 (0.00)	
	white	32038 (78.11)	0 (0.00)	
Sequence.number (%)	more	13557 (33.05)	252 (30.77)	0.181
	One primary only	27461 (66.95)	567 (69.23)	
Time (mean (SD))	not available	39.842 (30.760)	37.827 (30.885)	0.0634
status (%)	alive	30487 (74.33)	602 (73.50)	0.6224
	dead	10531 (25.67)	217 (26.50)	
Sex (%)	female	14530 (35.42)	299 (36.51)	0.5448
	male	26488 (64.58)	520 (63.49)	
Primary.Site (%)	C64.9-Kidney	39018 (95.12)	731 (89.26)	<0.0001
	C65.9-Renal pelvis	2000 (4.88)	88 (10.74)	
Grade (%)	Moderately differentiated	14234 (34.70)	306 (37.36)	<0.0001
	Poorly differentiated	8662 (21.12)	242 (29.55)	
	Undifferentiated; anaplastic	3245 (7.91)	68 (8.30)	
	unknown	11602 (28.29)	126 (15.38)	
	Well differentiated	3275 (7.98)	77 (9.40)	
Pathological (%)	8120/3	1082 (2.64)	33 (4.03)	0.0014
	8130/3	998 (2.43)	29 (3.54)	
	8260/3	5130 (12.51)	75 (9.16)	
	8310/3	21888 (53.36)	456 (55.68)	
	8312/3	7398 (18.04)	139 (16.97)	
	8317/3	2160 (5.27)	50 (6.11)	
	other (n<1000)	2362 (5.76)	37 (4.52)	
T (%)	T1	27118 (66.11)	497 (60.68)	0.0021
	T2	4108 (10.02)	98 (11.97)	
	T3	8098 (19.74)	180 (21.98)	
	T4	1061 (2.59)	21 (2.56)	
	TX	633 (1.54)	23 (2.81)	
Tumor.Size (mean (SD))	not available	51.355 (41.109)	51.877 (37.304)	0.7186

IQR, interquartilerange; Other, less than 1,000 cases.

**Table 2:** Baseline renal cancer patients with and without lymph node metastasis.

Characteristics	Level	NLMs (N=39141)	LMs (N=2696)	p
category (%)	Training group	38388 (98.08)	2630 (97.55)	0.0675
	Validation group	753 (1.92)	66 (2.45)	
Marital (%)	Married	23163 (59.18)	1517 (56.27)	<0.0001
	unknown	1916 (4.90)	86 (3.19)	
	unmarried	14062 (35.93)	1093 (40.54)	
Age (median [IQR])	not available	64.000 [55.000, 72.000]	66.000 [57.000, 76.000]	<0.0001
Race.ethnicity (%)	black	4918 (12.56)	307 (11.39)	0.1844
	Chinese	1215 (3.10)	96 (3.56)	
	other	3057 (7.81)	206 (7.64)	
	white	29951 (76.52)	2087 (77.41)	
Sequence.number (%)	more	13160 (33.62)	649 (24.07)	<0.0001
	One primary only	25981 (66.38)	2047 (75.93)	
times (mean (SD))	not available	41.480 (30.663)	15.446 (20.027)	<0.0001
status (%)	alive	30431 (77.75)	658 (24.41)	<0.0001
	dead	8710 (22.25)	2038 (75.59)	
Sex (%)	female	13956 (35.66)	873 (32.38)	0.0006
	male	25185 (64.34)	1823 (67.62)	
Primary.Site (%)	C64.9-Kidney	37455 (95.69)	2294 (85.09)	<0.0001
	C65.9-Renal pelvis	1686 (4.31)	402 (14.91)	
Grade (%)	Moderately differentiated	14373 (36.72)	167 (6.19)	<0.0001
	Poorly differentiated	8286 (21.17)	618 (22.92)	
	Undifferentiated; anaplastic	2679 (6.84)	634 (23.52)	
	unknown	10472 (26.75)	1256 (46.59)	
	Well differentiated	3331 (8.51)	21 (0.78)	
Pathological (%)	8120/3	783 (2.00)	332 (12.31)	<0.0001
	8130/3	936 (2.39)	91 (3.38)	
	8260/3	4972 (12.70)	233 (8.64)	
	8310/3	21526 (55.00)	818 (30.34)	
	8312/3	6774 (17.31)	763 (28.30)	
	8317/3	2168 (5.54)	42 (1.56)	
	other(n<1000)	1982 (5.06)	417 (15.47)	
T (%)	T1	27177 (69.43)	438 (16.25)	<0.0001
	T2	3808 (9.73)	398 (14.76)	
	T3	7075 (18.08)	1203 (44.62)	
	T4	612 (1.56)	470 (17.43)	
	TX	469 (1.20)	187 (6.94)	
Tumor.Size (mean (SD))	not available	48.881 (39.220)	87.434 (49.112)	<0.0001

NLMs, no lymph node metastasis; LMs, lymph node metastasis; Other, less than 1,000 cases.

### Independent Risk Factors for Lymphatic Metastasis

In order to determine the LMs-related variables of RCC patients, 16 variables were analyzed. We conducted univariate and multivariate logistic regression analysis to explore independent risk factors for lymphatic metastasis. First, through univariate regression analysis, 15 variables were found to be significantly associated with lymphatic metastasis. Subsequently, after conducting multivariate regression analysis, 11 variables: age, marriage, one primary only or more, liver metastasis, lung metastasis, M staging, T staging, tumor differentiation grade, pathological classification, and tumor size, were identified as independent prognostic factors for lymphatic metastasis in RCC patients (all *P* < 0.05, **Table 3**).

**Table 3:** Univariate and multivariate Logistic regression for lymphatic metastasis of renal carcinoma.

Characteristics	Univariate logistics			Multivariable logistics		
	OR	CI	P	OR	CI	P
Age	1.01	1.01-1.02	<0.001	1	0.99-1	0.022
Bone.metastases						
No	Ref	Ref	Ref	Ref	Ref	Ref
Yes	9.67	8.68-10.78	<0.001	1.07	0.93-1.23	0.34
Brain.metastases						
No	Ref	Ref	Ref	Ref	Ref	Ref
Yes	7.51	6.17-9.14	<0.001	0.91	0.73-1.14	0.41
Unknown	8.37	4.26-16.46	<0.001	0.93	0.43-2	0.845
Grade						
Well differentiated	Ref	Ref	Ref	Ref	Ref	Ref
Moderately differentiated	1.79	1.14-2.83	0.012	1.34	0.84-2.14	0.219
Poorly differentiated	11.31	7.3-17.5	<0.001	3.85	2.45-6.04	<0.001
Undifferentiated; anaplastic	36.45	23.53-56.48	<0.001	4.55	2.88-7.18	<0.001
unknown	18.53	12.01-28.57	<0.001	4.24	2.7-6.64	<0.001
Liver.metastasis						
No	Ref	Ref	Ref	Ref	Ref	Ref
Yes	16.35	14.26-18.75	<0.001	1.42	1.2-1.67	<0.001
Unknown	10.7	6.04-18.97	<0.001	1.12	0.57-2.2	0.744
M						
M0	Ref	Ref	Ref	Ref	Ref	Ref
M1	21.85	20-23.86	<0.001	7.37	6.31-8.61	<0.001
Marital						
Married	Ref	Ref	Ref	Ref	Ref	Ref
Unmarried	1.2	1.11-1.3	<0.001	1.11	1-1.22	0.047
Unknown	0.69	0.55-0.86	0.001	0.83	0.64-1.07	0.15
Pathological						
8310/3	Ref	Ref	Ref	Ref	Ref	Ref
8312/3	2.95	2.66-3.27	<0.001	2	1.75-2.28	<0.001
8260/3	1.23	1.05-1.43	0.008	2.76	2.32-3.29	<0.001
8317/3	0.5	0.36-0.69	<0.001	0.81	0.58-1.14	0.232
8120/3	11.02	9.5-12.79	<0.001	4.58	3.26-6.45	<0.001
8130/3	2.58	2.05-3.24	<0.001	1.99	1.32-2.99	0.001
other(n<1000)	5.52	4.86-6.27	<0.001	2.87	2.45-3.38	<0.001
Primary.Site						
C64.9-Kidney	Ref	Ref	Ref	Ref	Ref	Ref
C65.9-Renal pelvis	3.9	3.47-4.4	<0.001	1.52	1.08-2.13	0.015
Pulmonary.metastasis						
No	Ref	Ref	Ref	Ref	Ref	Ref
Yes	15.22	13.89-16.67	<0.001	1.21	1.05-1.4	0.007
Race.ethnicity						
White	Ref	Ref	Ref	Ref	Ref	Ref
Black	0.9	0.79-1.01	0.081	NA	NA	NA
Chinese	0.93	0.64-1.35	0.71	NA	NA	NA
Other	0.97	0.83-1.12	0.657	NA	NA	NA
Sequence number						
One primary only	Ref	Ref	Ref	Ref	Ref	Ref
more	0.62	0.57-0.68	<0.001	0.89	0.8-0.99	0.039
Sex						
Male	Ref	Ref	Ref	Ref	Ref	Ref
Female	0.86	0.79-0.94	0.001	0.96	0.87-1.07	0.475
T						
T1	Ref	Ref	Ref	Ref	Ref	Ref
T2	6.35	5.51-7.32	<0.001	2.53	2.14-2.99	<0.001
T3	10.42	9.3-11.67	<0.001	4.18	3.64-4.8	<0.001
T4	47.28	40.52-55.16	<0.001	6.7	5.53-8.11	<0.001
TX	25.7	21.14-31.26	<0.001	3.49	2.77-4.39	<0.001
Tumor.Size	1.02	1.02-1.02	<0.001	1.00	1.00-1.00	<0.001

OR, odds ratio; 95% CI, 95% confifidence interval.

### Construction and Validation of Nomogram

**Figure 1:**
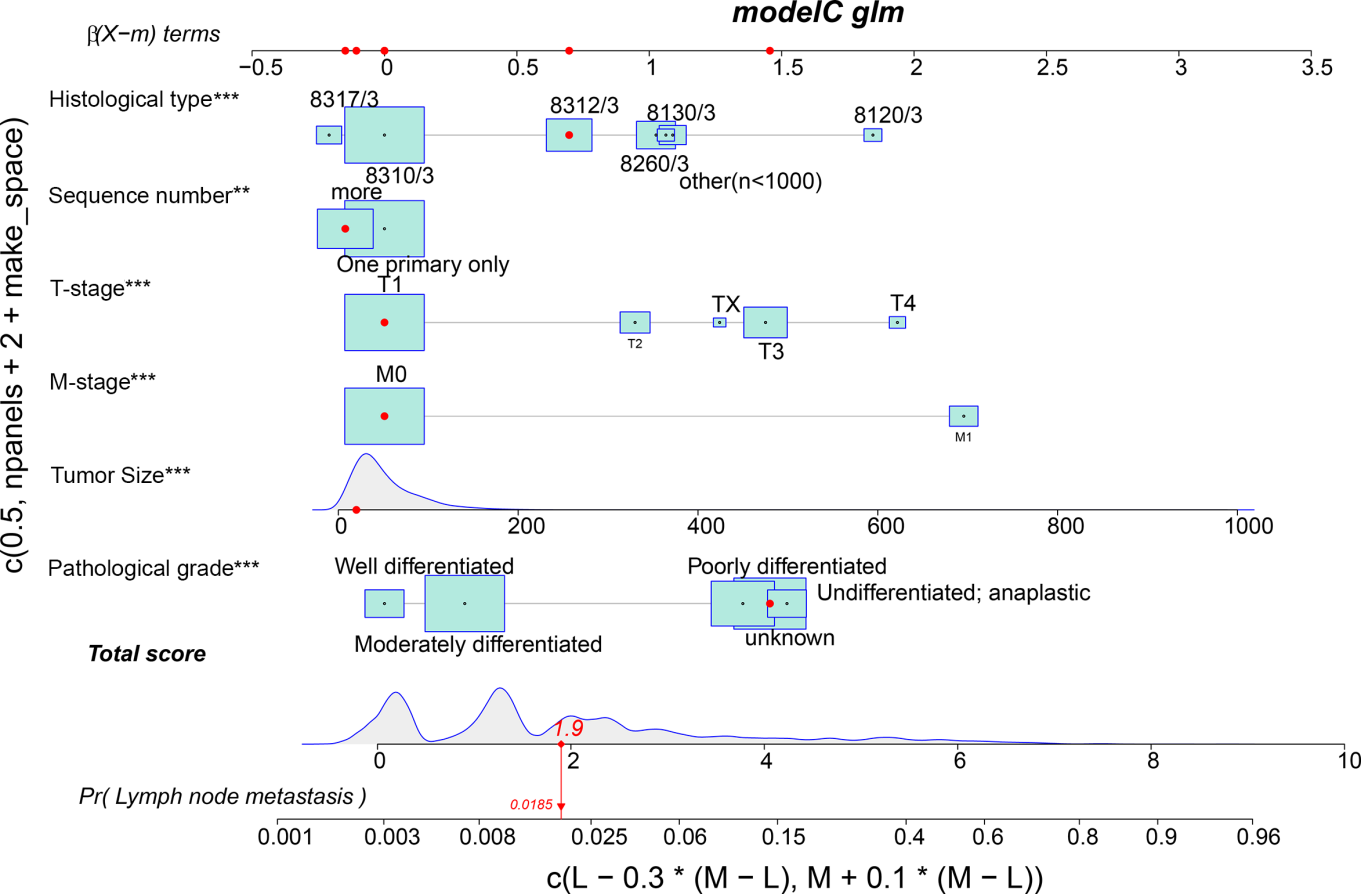
A nomogram for predicting the risk of lymphatic metastasis in patients with kidney cancer. 8317/3 represents chromophobe renal cell carcinoma, 8310/3 represents clear cell adenocarcinoma, 8312/3 represents renal cell carcinoma, 8260/3 represents papillary adenocarcinoma, 8130/3 represents papillary transitional cell carcinoma, 8120/3 represents transitional cell carcinoma, and other represents the number of patients is less than 1,000. Independent factors, **,<0.01; ***,<0.001.

**Figure 2:**
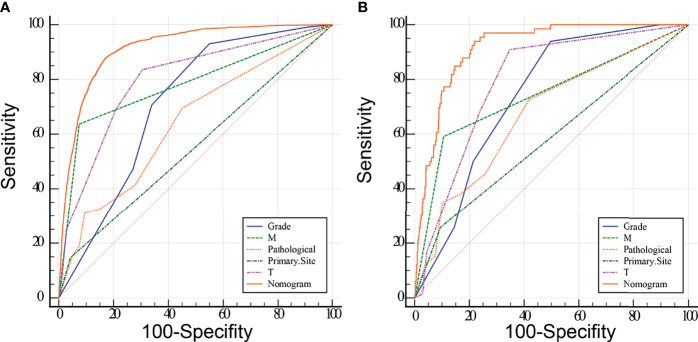
Receiver operating characteristic (ROC) analyses of the nomogram and each independent predictor based on the training **(A)** and validation **(B)** group. The results show that the nomogram has better predictive performance than any single variable.

**Figure 3:**
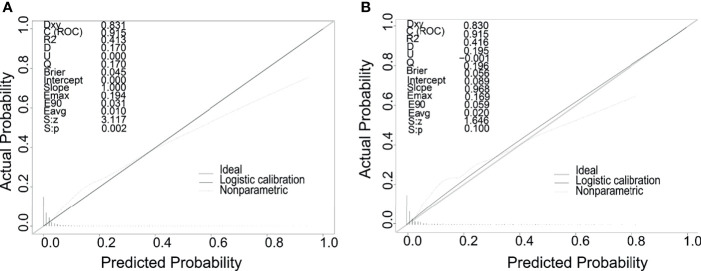
Calibration curves of the nomogram for predicting LMs in patients with RCC in the training cohort **(A)** and the validation cohort **(B)**. The x-axis represents the nomogram-predicted probability of LMs; the y-axis represents the actual probability of LMs.

**Figure 4:**
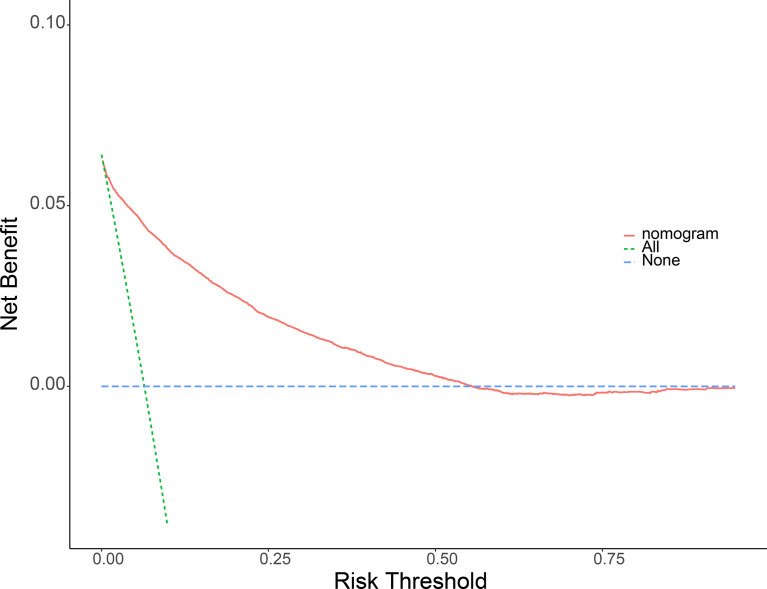
Decision curve analysis (DCA) of the nomogram for predicting LMs in patients with RCC in the training cohort **(A)** and the validation cohort **(B)**. The blue line represents the hypothesis that all RCC patients do not have lymphatic metastasis. The green line represents the hypothesis that all patients with RCC present lymphatic metastasis. The red line represents the nomogram. The y-axis represents net benefit, and the x-axis represents threshold probability. This diagnostic nomogram shows a notable positive net benefit, indicating that it has a good clinical utility in predicting estimating the risk of LMs in patients with RCC.

**Figure 5:**
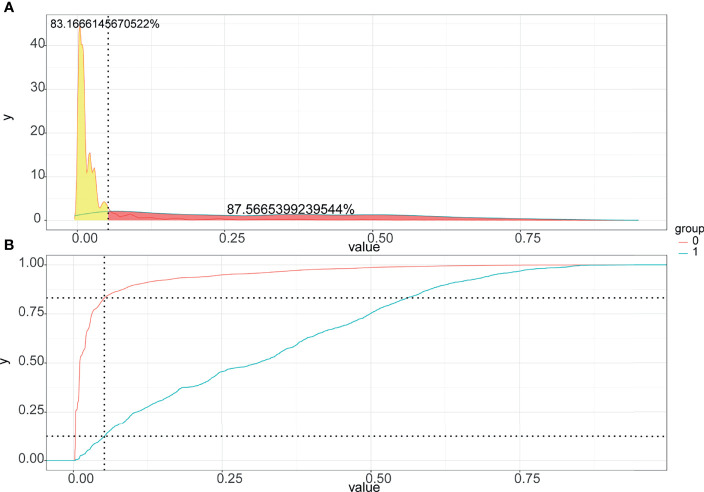
Probability density function graph **(A)** and Clinical utility curve **(B)** of the nomogram.

**Figure 6:**
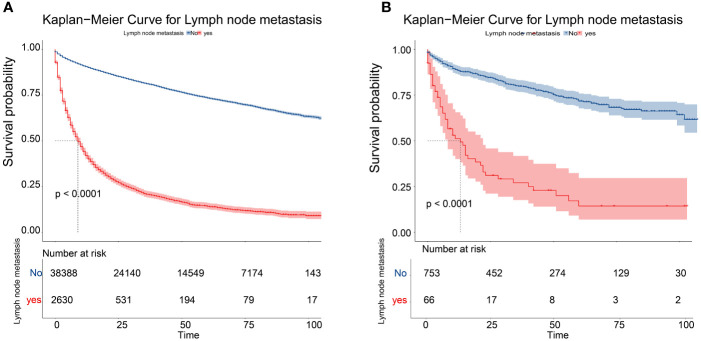
Kaplan–Meier overall survival curves of patients with LMs or not in the training cohort **(A)** and the validation cohort **(B)**.

Meaningful clinical indicators after multivariate analysis were included in the constructing a nomogram (**Figure 1**), including: pathological subtype, single/multiple tumors, tumor T, M staging, differentiation grade, and tumor size. In the nomogram, the values of specific patients were positioned along each variable axis, and a vertical line was drawn up to the dot axis to obtain the score for each variable. The score of each variable was added to get the total score, which was displayed on the total score line at the bottom of the nomogram. Then we would get the probability by drawing a vertical line from the total score to the LMs axis. In order to evaluate and verify the nomogram, the ROC curve of each independent LMs-associated risk factor was drawn in **Figure 2**. The AUC of the training group and the validation group reached 0.916, with 95% CI (0.913 to 0.918) and (0.895 to 0.934) respectively, indicating that the risk model possessed excellent discriminative ability (**Table 4**). What’s more, it showed the univariant association and the discrimination power measured by the AUC for each predictor variable in the training and verification groups (**Table 4**). As shown in **Figure 3**, the calibration chart verified that the predictive ability of the nomogram in the training group was highly consistent with the actual results. The results of DCA indicated that the nomogram had a significant positive net benefit in the process of predicting risk, confirming its good clinical application value (**Figure 4**). The probability density function (PDF) showed that the distribution of the nomogram probability in non-metastatic patients was sharply clustered, while the distribution in metastatic patients was relatively flat (**Figure 5**). Clinical utility curve (CUC), as a means to assist the translation of model information to the clinician, was used for determining the optimal prediction score threshold for each subgroup. For example, it showed that under the same threshold, the percentage of non-metastatic patients and metastatic patients could be detected (**Figure 5**). Furthermore, in order to assess the effect of lymphatic metastasis on the OS of RCC patients, we performed Kaplan-Meier survival analysis in the two groups of patients. As shown in **Figure 6**, whether in the training group or in the validation group, the OS of different lymph node metastasis status was significantly different (P < 0.0001), and the survival rate of patients without lymph node metastasis was significantly higher than that of patients with lymph node metastasis. In addition, we created a network calculator (https://liwenle0910.shinyapps.io/DynNomapp/) using independent risk factors obtained from the previous analysis, which could quickly and easily obtain the probability of lymph node metastasis in RCC patients.

**Table 4:** Area under the ROC Curve (AUC) of the training and verification groups.

Variable	AUC	SE	95% CI	AUC	SE	95% CI
Grade	0.713	0.00384	0.708 to 0.717	0.74	0.0229	0.708 to 0.769
M	0.781	0.00474	0.777 to 0.785	0.742	0.031	0.711 to 0.772
Pathological	0.64	0.00542	0.635 to 0.644	0.673	0.0334	0.640 to 0.705
Primary.Site	0.552	0.00348	0.547 to 0.557	0.582	0.0276	0.547 to 0.616
T	0.799	0.00429	0.796 to 0.803	0.794	0.0231	0.765 to 0.821
Nomogram	0.916	0.00251	0.913 to 0.918	0.916	0.0133	0.895 to 0.934

SE, standard error; 95% CI, 95%, confifidence interval.

## Discussion

RCC is a deadliest malignant urinary system tumor with high tumor heterogeneity and high recurrence rate (15), especially clear cell renal cell carcinoma (ccRCC) (16). Studies have shown that about 25% of patients with RCC have metastasised at the time of diagnosis, and 35% of them will develop distant metastases (DMs) during the process of tumor progression, resulting in a 5-year survival rate dropping about 10% (17). Due to resistance to chemotherapy and hormone therapy, surgical resection is still the main treatment for RCC at present. Considering the patient’s pathology and lymphatic metastasis, clinicians are often confronted with the difficulty of selecting surgical methods and scope (18). However, there are still quite a few patients undergoing recurrence and metastasis after surgery, which makes it difficult to accurately predict the survival rate of RCC patients. In recent years, with the advent of targeted therapy, median survival for metastatic kidney cancer has roughly doubled. Furthermore, immunotherapy based on anti-PD-1/PD-L1 inhibitors has been shown to be more effective than sunitinib in the first-line treatment of advanced renal cell carcinoma (RCC). To our knowledge, sarcomatoid RCC (srRCC) is prone to metastases with poor prognosis and limited treatment options. A systematic review and meta-analysis found out that sarcomatoid histology might be associated with improved response to PD-1/PDL-1 compared with sunitinib (19). TNM staging is an excellent cancer staging and prognostic system and is determined by the primary tumor stage (the size and extent of tumor expansion), lymph node metastasis and distant metastasis (20, 21). Lymph node metastasis doubles the risk of distant metastasis in patients and has a significant negative impact on the progression-free survival and overall survival of patients with metastatic RCC (15). With the rapid development of multiple imaging methods, the identification and detection of lymph node metastasis have improved, but micrometastasis is often overlooked. Therefore, exploring LMs-related predictors and identifying RCC patients with high risk of LMs seem to be of great significance for clinical decision-making and personalized management.

RCC is a type of tumor with gender-biased characteristics (22). According to statistics, the number of cases in men is almost twice that of women. Compared with women, male RCC patients show poorer initial tumor characteristics and higher cancer-specific mortality and worse disease outcomes after surgical treatment (23). Miki et al. analyzed the differences in age and gender of RCC, and the results showed that women had an older age of RCC then man, but the tumor stage and size were smaller than men (24). Smoking and drinking have been widely recognized as independent risk factors for RCC (22), and these behaviors are mostly found in males, so the incidence of male patients is higher. A study on androgen receptor (AR) overexpression increased blood metastasis but reduced LMs showed that there was also a gender difference between lung metastasis and lymph node metastasis in RCC patients. The results suggested that if the AR was overexpressed, RCC was more likely to metastasize to the lung, and conversely, it was more prone to LMs (25).

The occurrence of RCC is related to a variety of gene mutations and exposure to environmental risk factors (22). Our nomogram showed that the most common pathological subtype of RCC was ccRCC. The protein-coding mutations for ccRCC have been widely characterized, involving the inactivation of Von Hippekl Lindau (VHL) tumor suppressor (16), and the induction of HIF and VEGF. The metastasis of RCC mainly occurs through hematogenous and lymphatic pathways, and the occurrence of these two types of metastases is related to different microvessel density and angiogenesis-specific factors. Among them, the most common site of blood-borne metastasis is the lung. Studies shown that when RCC patients developed LMs, VEGF-C increased, and VEGF-A decreased, When PM occurred, VEGF-A increased, and VEGF-C decreased (25). According to reports, the appearance of PM was significantly related to the difference in progression-free survival (26). Zhang et al. discovered and characterized 17 ccRCC key metastasis-associated genes (MAGs) through single-cell sequencing and found that the increase in MAGs scores was associated with higher T staging, higher lymph node positive rate, late metastasis, poor pathological staging, and tumor grade. Finally, four independent risk factors related to RCC metastasis were determined, including age, tumor grade, pathological stage, and MAG score (27). Our findings were consistent with increasing evidence that the presence of metastasis predicted a worse clinical outcome.

The distribution and drainage of lymph nodes around the kidney are complex and cumbersome, and the existing imaging techniques are still very limited in the ability to identify LMs early. Lymph vessels and lymph nodes are mainly distributed around the veins. Given that lymphatic distribution is closely related to the course of the intrarenal veins, venous infiltration and lymphatic infiltration are inseparable from the LMs (28, 29). Venous infiltration is common in advanced RCC. It is not only an independent prognostic indicator of patient survival, but also a predictor of recurrence after radical surgery. By using immunohistochemistry to study the relationship between LMs and lymphatic invasion and lymphatic proliferation, it was found that tumor size, tumor cell type, tumor growth pattern, venous invasion, lymphatic invasion, and primary tumor stage were all related to LMs. Ultimately, lymphatic invasion was found to be an independent predictor of LMs in RCC. Moreover, it was considered that the expansion of the tumor and proliferation of lymph nodes around the tumor may increase the chance of tumor cells leaving the primary site (30). It was in line with the risk factors of LMs in RCC patients found in our research.

Radical nephrectomy is the main treatment for RCC. Ideally, kidney disease and lymph nodes in the lymphatic drainage area must be removed, which is one of the important conditions for curing (31). Although LMs is a major factor in determining the clinical stage and predicting the prognosis of patients, there are still controversies about the role of extensive lymphadenectomy in the surgical treatment of RCC and whether it affects the survival of patients. A prospective randomized controlled trial evaluated whether complete lymph node dissection combined with radical nephrectomy was more effective than radical nephrectomy alone. The results could not prove the survival advantage of complete lymph node dissection combined with radical nephrectomy, which might be due to the low incidence of unexpected LMs after proper preoperative staging (4.0%) (32). Regardless of the fact that the incidence of LMs is low, in some studies, lymph node involvement has been determined by some studies as an independent risk factor for poor tumor prognosis, and it still needs our attention.

Predicting risks of LMs in RCC patients is crucial for the patient’s prognostic consultation. It is also of valuable significance in designing clinical trials, evaluating the clinical results, patient psychological counseling, and programmed management and treatment. Medical nomogram is a model that uses biological and clinical variables (such as tumor grade and patient age) to graphically describe statistical prognosis and generates the probability of occurrence of individual patients’ clinical events (such as cancer recurrence or death), which is widely used in various malignant tumors. Additionally, radiomics and genomics have shown great promise in cancer research, such as improving risk stratification and disease management in prostate cancer (PCa) patients. In the near future, it is also hoped that it can be applied to kidney cancer (33).

Based on the information of patients diagnosed as RCC in the SEER database, this study constructed a nomogram to quantify the risk of LMs in RCC patients and verified it in the patient population from the Second Affiliated Hospital of Dalian Medical University. The total score obtained by combining different risk factors predicted the probability of developing LMs in RCC patients. The higher the score, the higher the risk of LMs. The ROC and AUC analysis showed that the nomogram had excellent predictive ability. The calibration chart indicated that the nomogram had a high degree of consistency in prediction and practical applications. DCA showed that the predictive model had a significant positive net benefit in its application. All these results showed that these independent risk factors were well in predicting LMs in patients with RCC, not only in the training group, bus also in the validation group. Although the verification group is small, it can be well verified on the results of the training group. In addition, we will continue to collect more clinical data for Prospective research. Additionally, in conjunction with the risk factors that played an important role, we also created a web calculator (https://liwenle0910.shinyapps.io/DynNomapp/) to help clinicians easily and quickly obtain the probability of LMs in RCC patients. Overall, our nomogram may be the first useful method for accurately predicting LMs in patients with RCC to date. However, as a retrospective analysis, there were several limitations in our study, including selection bias, information bias, lack of standardization of diagnosis, treatment and follow-up, missing or unavailability of some information (such as smoking, drinking history), tumors markers, etc. Despite these limitations, our nomogram was based on a large number of samples, and internal and external verification to ensure the credibility. In the future, a more complete experimental design will be needed to facilitate clinical application.

## Conclusion

Through retrospective analysis of RCC patient information from the SEER database and the Second Affiliated Hospital of Dalian Medical University, we obtained the independent factors for lymph node metastasis in patients with RCC. By integrating these factors, we constructed a nomogram to predict the risk of lymphatic metastasis in RCC patients. After drawing a series of verification curves, it was confirmed that the nomogram had good calibration and discrimination. The poor prognosis of LMs patients was confirmed by Kaplan-Meier curve. Moreover, a web version of the nomogram, a simple network calculator, had likewise been established to facilitate clinical application. The nomogram we made can uniquely, conveniently, and intuitively quantify the risk of LMs in RCC patients, and then guide clinicians to predict prognosis and make individualized treatment decisions for patients.

## Data Availability Statement

The raw data supporting the conclusions of this article will be made available by the authors, without undue reservation.

## Author Contributions

CY, MH carried out the study design. WL, BW and SD conducted the research and collected and analyzed the data. WL performed the statistical analysis and drafted the manuscript. XQ and XX provided the expert consultations and suggestions. All conceived the study, participated in its design and coordination, and helped shape the language. All authors contributed to the article and approved the submitted version.

## Conflict of Interest

The authors declare that the research was conducted in the absence of any commercial or financial relationships that could be construed as a potential conflict of interest.

## Publisher’s Note

All claims expressed in this article are solely those of the authors and do not necessarily represent those of their affiliated organizations, or those of the publisher, the editors and the reviewers. Any product that may be evaluated in this article, or claim that may be made by its manufacturer, is not guaranteed or endorsed by the publisher.
